# Effect of Aromatic Substitution of Curcumin Nanoformulations on Their Stability

**DOI:** 10.3390/scipharm84040625

**Published:** 2016-04-19

**Authors:** Siriporn Okonogi, Ornchuma Naksuriya, Suporn Charumanee, Jakkapan Sirithunyalug

**Affiliations:** Department of Pharmaceutical Sciences, Faculty of Pharmacy, Chiang Mai University, Suthep Road, Chiang Mai 50200, Thailand; ornchuma77@gmail.com (O.N.); chsuporn@gmail.com (S.C.); jakkapan.s@cmu.ac.th (J.S.)

**Keywords:** curcumin, stability, polymeric micelles, nanoformulations

## Abstract

Curcumin, a poorly water-soluble bioactive compound, was successfully loaded into three different aromatic contents of hydroxypropylmethacrylamide (HPMA)-based polymeric micelles in order to develop water-soluble curcumin nanoformulations (Cur-Nano). The stability study of Cur-Nano was done by keeping the formulations at 4, 30, and 40 °C for 90 days. The physical appearance, curcumin remaining, and particle size of Cur-Nano were examined by visual inspection, high-performance liquid chromatography, and dynamic light scattering, respectively. After the storage period, the Cur-Nano composed of 100% aromatic-substituted polymer exhibited the highest stability of curcumin (80% of curcumin remaining) with a similar particle size as measured on the first day (50–60 nm) in all storage conditions. Curcumin in Cur-Nano composed of 25% and 0% aromatic-substituted polymer was significantly less stable accordingly. The results suggested that aromatic substitution to HPMA-based polymeric micelles can significantly enhance the stability of the loaded curcumin, considerably due to the π-π stacking interactions between the aromatic groups of curcumin and the polymer. It is concluded that curcumin-loaded polymeric micelles with high substituted aromatic content can be promising candidates with good storage stability for further clinical evaluations.

## 1. Introduction

**Figure 1:**
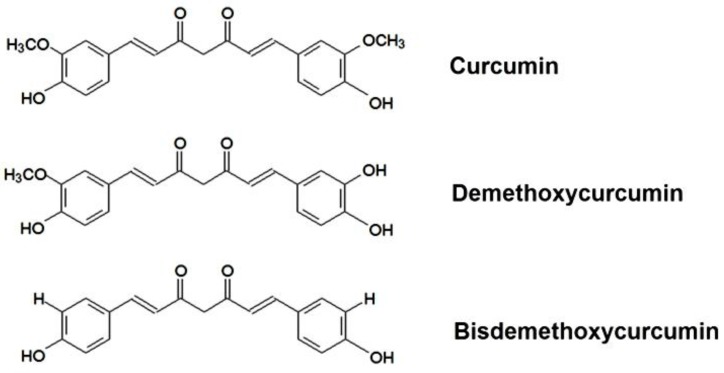
Chemical structure of curcumin and its derivatives.

Curcumin ([1,7-bis(4-hydroxy-3-methoxyphenyl)-1,6-heptadiene-3,5-dione]) is a natural yellow-colored phenolic antioxidant. Curcumin from different natural sources as well as that available as a commercial product is isolated from the rhizome of *Curcuma longa* L. in which it presents in relatively high concentrations. The commercially available product contains 77% of curcumin and its two derivatives (17% of demethoxycurcumin and 3% of bisdemethoxycurcumin) which have similar chemical structures, as seen in Figure 1. Curcumin demonstrates various health benefits against many chronic diseases including diabetes, inflammatory diseases, infectious diseases, liver diseases, Alzheimer’s disease, and cancers [1,2,3,4,5]. Although curcumin possesses many beneficial properties, its low aqueous solubility and poor stability remain major barriers for clinical efficacy. Orally administered curcumin is mostly excreted in faeces and urine after rapid metabolism [6]. It was reported that curcumin solubility in aqueous buffer (pH 5.0) was only 11 ng/mL [7]. Furthermore, only 0.6 μmol/L of free curcumin and its conjugates were detected in the rat’s serum [8]. Another reason that limits clinical application of curcumin is that curcumin degrades quickly in neutral or alkaline buffer solution [9].

In recent years, nanotechnology has been applied to pharmaceutical fields to develop novel dosage forms as nanoformulations. These systems alter the pharmacokinetics, biodistribution, stability, solubility, and drug release of associated drugs [10]. Additionally, they show a low immunogenicity and reduce unwanted side effects [11]. In the present study, nanotechnology using polymeric micellization was applied to overcome curcumin limitations as to enhance its solubility and stability.

Poly [*N*-(2-hydroxypropyl) methacrylamide] (pHPMA) is a hydrophilic polymer currently used for pharmaceutical and biomedical applications because of its biocompatibility, non-immunogenicity, and its possibilities for chemical functionalization [12,13,14,15]. In previous studies, nanoformulations of HPMA-based polymers have been used to solubilize hydrophobic molecules like paclitaxel [16], docetaxel [17], and xanthone [18]. It has also been recently reported that curcumin-loaded HPMA-based polymeric micelles showed excellent loading and retention as well as an enhanced inhibitory effect against various cancer cell lines [19]. However, a successful formulation requires not only efficacy but also stability during storage. In the present study, block copolymers of polyethylene glycol (PEG) and HPMA monomers with different percentages of aromatic contents (in parenthesis) of which were *N*-(2-hydroxypropyl) methacrylamide dilactate (HPMA-DL) (0%), *N*-(2-benzoyloxypropyl) methacrylamide with *N*-(2-hydroxypropyl) methacrylamide monolactate (HPMA-BZ-ML) (25%), and *N*-(2-benzoyloxypropyl) methacrylamide (HPMA-BZ) (100%), were used to form polymeric micelle nanocarriers in order to entrap curcumin and fabricate a curcumin nanoformulation, a so-called “Cur-Nano”. The obtained nanoformulations were evaluated for their stability under various storage temperatures. The outer appearance, precipitation, amount of curcumin remaining, particle size, and polydispersity index (PdI) of Cur-Nano were investigated during the storage period.

## 2. Materials and Methods

### 2.1. Chemicals

ω-Methoxy poly(ethylene glycol)-b-(*N*-(2-hydroxypropyl) methacrylamide dilactate) (PEG-HPMA-DL), ω-methoxy poly(ethylene glycol)-b-(*N*-(2-benzoyloxypropyl) methacrylamide)-co-(*N*-(2-lactoyloxypropyl) methacrylamide) (PEG-HPMA-BZ-ML), and ω-methoxy poly(ethylene glycol)-b-(*N*-(2-benzoyloxypropyl) methacrylamide) (PEG-HPMA-BZ) were synthesized and characterized as described previously [19]. The average molecular weight (Mw) of PEG-HPMA-DL, PEG-HPMA-BZ-ML, and PEG-HPMA-BZ as determined by gel permeation chromatography (GPC) were 27, 29, and 28 kDa, respectively. Curcumin was purchased from Sigma-Aldrich (St. Louis, MO, USA) (No. 28260). Acetone, acetonitrile, ammonium acetate, acetic acid, and methanol were purchased from Merck, Darmstadt, Germany. All other chemicals and solvents were of the highest grade available.

### 2.2. Preparation and Characterization of Cur-Nano

Curcumin-loaded PEG-HPMA-DL micelles (Cur-Nano1), curcumin-loaded PEG-HPMA-BZ-ML micelles (Cur-Nano2), and curcumin-loaded PEG-HPMA-BZ micelles (Cur-Nano3) were prepared as described previously [19,20]. In detail, 50 mg of PEG-HPMA-DL polymers and PEG-HPMA-BZ-ML were separately dissolved in 4.5 mL of ammonium acetate buffer pH 5.0 and incubated at 0–4 °C for 16 h. A stock solution of 10 mg/mL of curcumin in acetone (1 mL) was added to the polymer solutions at 0–4 °C. Then the mixture was immediately heated in a water bath (50 °C) for 1 min to form polymeric micelles. The mixture was slowly cooled down to room temperature. For the Cur-Nano3 preparation, 100 mg of PEG-HPMA-BZ polymer was dissolved in 1 mL of acetone, and a stock solution of 10 mg/mL of curcumin in acetone (0.5 mL) was mixed to the polymer solution. The mixture was added dropwise to 5 mL of ammonium acetate buffer pH 5.0 while the buffer was stirred and it was continuously stirred for 2 h allowing the acetone to evaporate. After that, all Cur-Nano was separately passed through a 0.22 μm filter. The concentration of curcumin in Cur-Nano was determined by adding 100 μL of Cur-Nano to 900 μL of methanol and analyzing it by reversed-phase high-performance liquid chromatography (RP-HPLC). The encapsulation efficiency (EE) and loading capacity (LC) were calculated as follows:

$$\mathrm{EE}=\frac{\mathrm{amount of loaded curcumin}}{\mathrm{amount of curcumin used for loading}}\times 100$$

and

$$\mathrm{LC}=\frac{\mathrm{amount of loaded curcumin}}{\mathrm{amount of copolymer used for loading}}\times 100$$

### 2.3. Reversed-Phase High-Performance Liquid Chromatography (RP-HPLC)

HPLC analysis was carried out on an Agilent system (Santa Clara, CA, USA) using an analytical C18 column, (SunFire, Waters Corporation, Milford, MA, USA) (5 µm, 150 × 4.6 mm). A gradient system was run from 5:95 (*v*/*v*) acetonitrile/water as eluent A and acetonitrile as eluent B. Both eluents were adjusted by the addition of 0.25% acetic acid. The gradient was run from 45% A to 60% B in 10 min. The injection volume was 20 µL, the flow rate was 1.2 mL/min, and the detection wavelength was 425 nm.

### 2.4. Stability Test under Storage Conditions

All three Cur-Nano formulations (2 mL) were stored in closed containers at various temperatures of 4, 30, and 40 °C for 90 days. Changes in physical appearance were investigated visually. At different time points, Cur-Nano aliquots of 100 µL were withdrawn, added to 900 µL of methanol, and the curcumin content was analyzed by RP-HPLC.

### 2.5. Particle Size Determination of Cur-Nano

Cur-Nano (20 µL) was diluted in deionized water (980 µL). The Z_ave_ particle size and size distribution of polymeric micelles were measured by using a Zetasizer ZS (Malvern Instruments, Ltd., Malvern, UK). The size measurements were taken at a fixed angle of 173°.

## 3. Results and Discussion

### 3.1. Characterization of Cur-Nano

**Figure 2:**
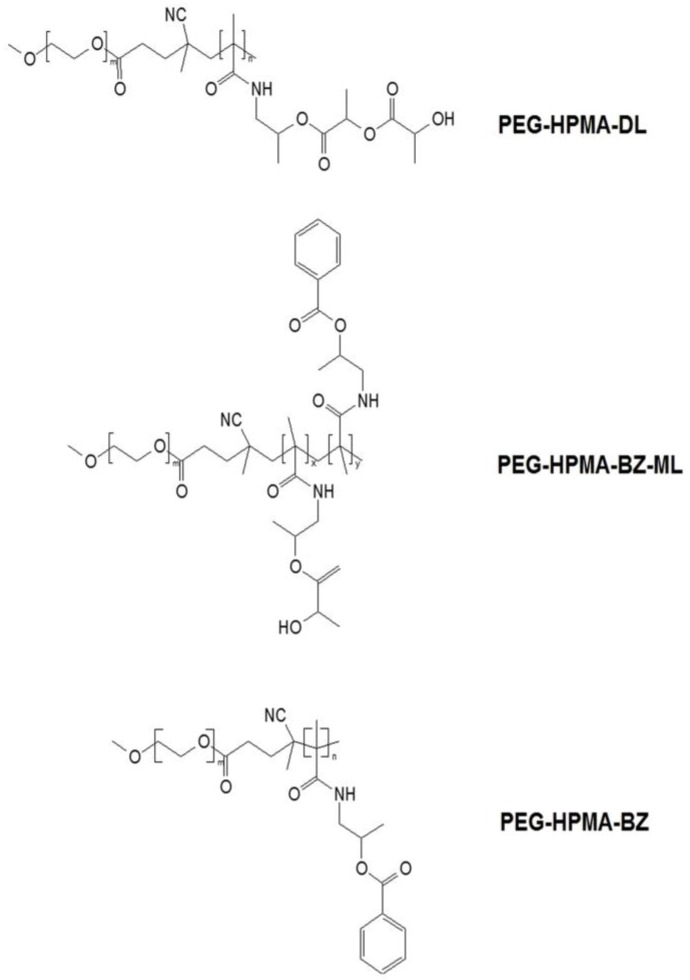
Chemical structures of the three hydroxypropylmethacrylamide (HPMA)**-**based polymers. PEG: polyethylene glycol; HPMA-DL: *N*-(2-hydroxypropyl) methacrylamide dilactate; HPMA-BZ-ML: *N*-(2-benzoyloxypropyl) methacrylamide with *N*-(2-hydroxypropyl) methacrylamide monolactate; HPMA-BZ: *N*-(2-benzoyloxypropyl) methacrylamide.

Curcumin was successfully loaded into different aromatic content HPMA-based polymers (Figure 2), PEG-HPMA-DL (0% aromatic substitution), PEG-HPMA-BZ-ML (25% aromatic substitution), and PEG-HPMA-BZ (100% aromatic substitution). The appearance of the aqueous mixtures of all Cur-Nano was a clear orange-yellow color, whereas that of intact curcumin in the same amount was an opaque mixture suspended with the undissolved curcumin. This result obviously indicated that the aqueous solubility of curcumin was greatly enhanced by Cur-Nano. The EE and LC of Cur-Nano was different in each nanoformulation. Cur-Nano3 displayed the highest value of EE and LC of 113.8% ± 3.4% and 12.6% ± 0.4%, respectively, after loading 1 mg/mL of curcumin into 9 mg/mL of polymer as presented in Table 1. The results demonstrated that the EE and LC of Cur-Nano1 and Cur-Nano2 were significantly lower than that of Cur-Nano3 accordingly.

According to these results, it was calculated that curcumin was solubilized up to 600–1000 μg/mL in these water-soluble polymeric micellar systems. The water solubility of curcumin entrapped in these polymeric micelles can be enhanced up to approximately 5 × 10^4^–9 × 10^4^ times higher than that of curcumin intact in aqueous buffer pH 5.0. Cur-Nano3, which possessed 100% aromatic substitution, exhibited the highest solubility enhancement of curcumin. The result was considered to be due to the hydrophobic interaction between curcumin and the hydrophobic chain containing the aromatic ring of the polymers. The results showed that the higher the substituted aromatic content, the higher the curcumin solubility increased.

### 3.2. Curcumin Stability under Storage Conditions

**Figure 3:**
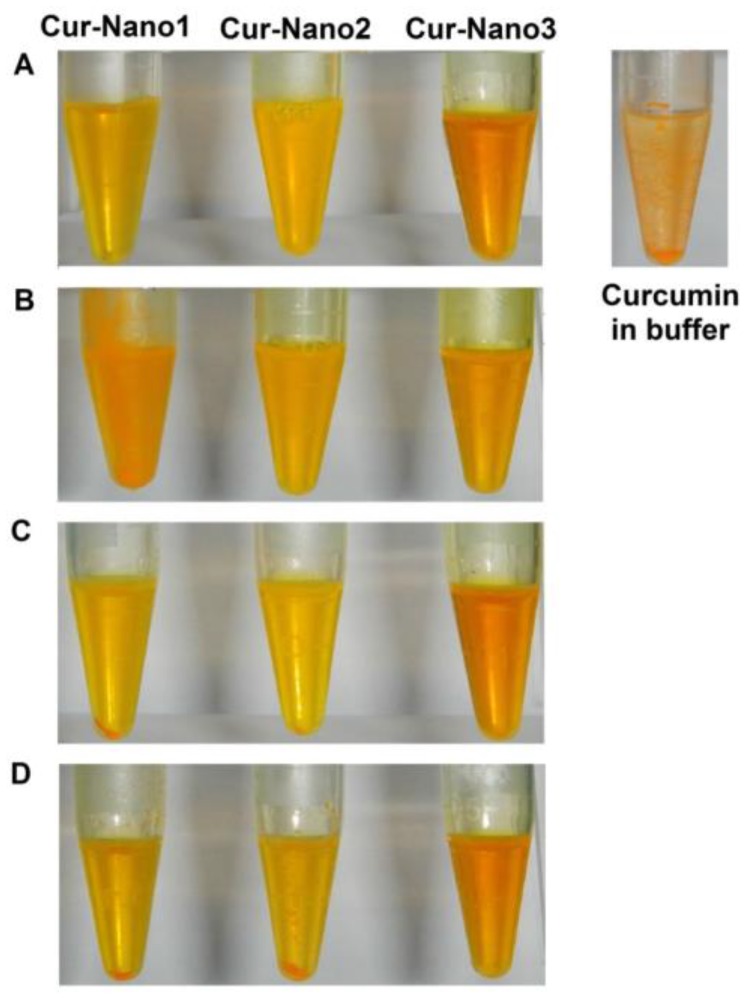
Physical appearance of curcumin in buffer compared to Cur-Nano at day 1 (**A**); physical appearance of Cur-Nano after 90 days at 4 (**B**); 30 (**C**); and 40 °C (**D**).

The stability study was carried out with different storage temperatures (4, 30, and 40 °C) in order to investigate curcumin stability after entrapment into polymeric micelles and suitable storage conditions of each Cur-Nano. After 90 days, the changes in physical appearance of the three Cur-Nano systems were observed as shown in Figure 3. At day 1, all formulations did not show any precipitation when compared to curcumin in ammonium acetate buffer pH 5.0. The amount of precipitation of curcumin that occurred in Cur-Nano1 and Cur-Nano2 kept at 40 °C was higher than that kept at 30 °C. Cur-Nano2 did not show precipitated curcumin when stored at 4 °C, whereas Cur-Nano1 revealed precipitation at 4 °C. Interestingly, Cur-Nano3 did not show any precipitation at 30 and 40 °C but some precipitation was observed after keeping it at 4 °C. In addition to the results from the physical appearance observation, it was in good agreement that Cur-Nano3 possessed the best stabilization of curcumin.

**Figure 4:**
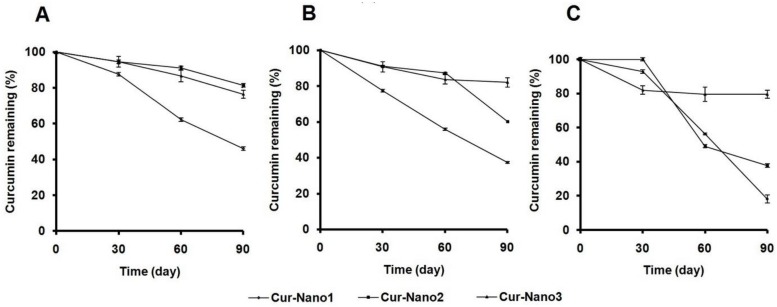
Percentage of curcumin remaining versus storage time of Cur-Nano at 4 (**A**); 30 (**B**); and 40 °C (**C**) (*n* = 3).

To confirm the stabilization effect by polymeric micelles, the amount of curcumin remaining in polymeric micelles was investigated. Figure 4A demonstrates that by keeping it at 4 °C for 90 days, curcumin content in polymeric micelles of Cur-Nano1 was lower (46% ± 1%) than that of Cur-Nano2 (80% ± 1%) and Cur-Nano3 (76% ± 2%). The amount of Cur-Nano1 decreased to 37% ± 1%, whereas Cur-Nano2 and Cur-Nano3 showed higher curcumin remaining with values of 60% ± 0% and 82% ± 3%, respectively, after incubation at 30 °C for 90 days (Figure 4B). Interestingly, after keeping it at 40 °C for 90 days, Cur-Nano3 revealed the highest amount of remaining curcumin, up to 80% ± 2%, while curcumin in Cur-Nano1 and Cur-Nano2 decreased to 18% ± 2% and 38% ± 1%, respectively. It was indicated that the stability of curcumin increased with an increase in aromatic content of the polymers. This finding was in line with a previous study which explained that the introduction of aromatic content can increase the stability of hydrophobic drugs in polymeric micelles [17].

### 3.3. Particle Size Determination of Cur-Nano

**Figure 5:**
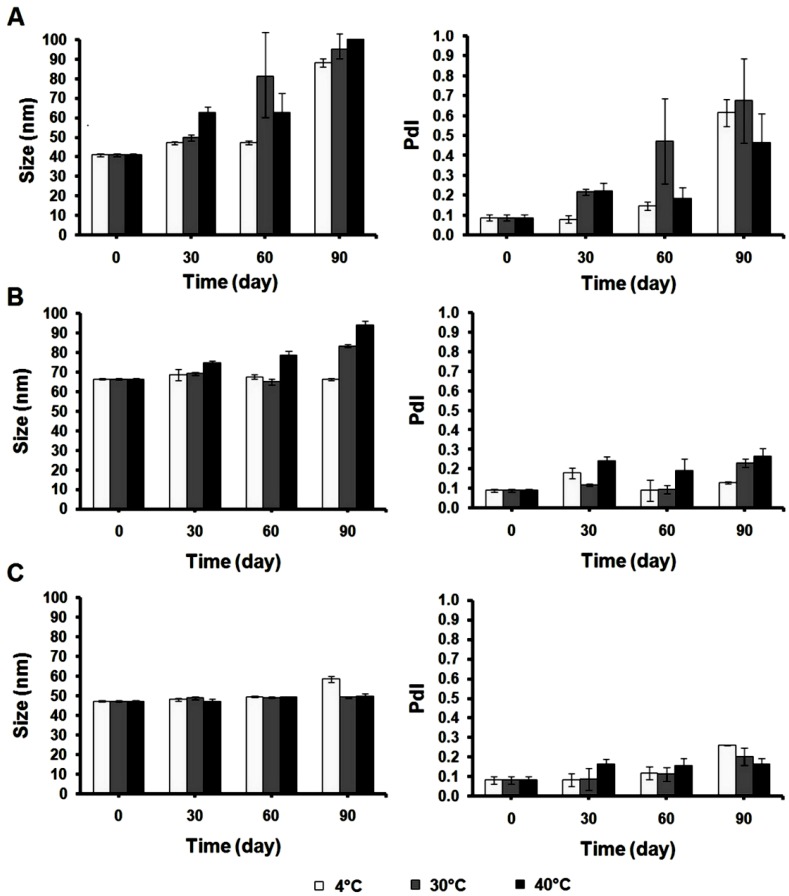
The particle size (left side) and polydispersity index (PdI) (right side) of Cur-Nano1 (**A**); Cur-Nano2 (**B**); and Cur-Nano3 (**C**) at different storage conditions (*n* = 3).

The particle sizes of all nanoformulations were examined after 30, 60, and 90 days of storage at 4, 30, and 40 °C. The results showed that the particle size of Cur-Nano1 increased significantly from 41 ± 1 nm to 88 ± 2, 95 ± 5, and 663 ± 233 nm after storage for 90 days at 4, 30, and 40 °C, respectively (Figure 5). Moreover, the size distribution expressed as the PdI of the systems was also increased. It was reported that after hydrolysis of the lactate side chain, the hydrophilicity of the polymer increased [21]. The result of the present study was in good agreement with this report that the higher hydrophilicity was obtained after long-term storage of Cur-Nano in an aqueous system and subsequently, swelling of the polymeric micelles. After consideration of storage temperature and time, it was found that the suitable storage condition of Cur-Nano1 was 4 °C for 30 days.

The particles of Cur-Nano2 retained a similar size at 4 °C (66 ± 1 nm) after 90 days, whereas the particle size at 30 and 40 °C increased to 84 ± 1 and 94 ± 2 nm, respectively, with the increase in PdI. The increased size of Cur-Nano2 was caused by the hydrolysis of the lactate side group of HPMA resulting in the hydration and swelling of the nanoparticles. This behavior was similar to Cur-Nano1, but the swelling of Cur-Nano2 was slower than Cur-Nano1 because of the stacking interactions of the aromatic unit. In addition, it is noted that Cur-Nano2 exhibited its stability at 4 °C even after long-term storage of 90 days, indicating that 4 °C is the suitable temperature for this system. Cur-Nano3 showed the smallest size of 50 ± 1 nm. Interestingly, Cur-Nano3 did not show an increase in particle size after incubating at 30 °C and 40 °C for the long period of 90 days. This system also showed a narrow size distribution with a PdI less than 0.2. However, the particle size and PdI of this system increased to 59 ± 2 nm and 0.3 ± 0.0, respectively, after 90 days at 4 °C. Moreover, some precipitation was observed in this system. This result suggested that the suitable storage temperature of Cur-Nano3 should be higher than 4 °C or at approximately 30–40 °C. It was previously reported that the benzene rings of aromatic-substituted HPMA polymers and the phenolic rings of curcumin can redistribute the π electrons over the rings causing the stacking interactions which influence the stability of the drug in the core of micelles [20,22,23]. As the polymer used in Cur-Nano3 was comparatively 100% aromatic substitution, it was therefore considered that the high stability in size and small size distribution of Cur-Nano3 were due to the π-π stacking interaction between the aromatic groups of curcumin and the used polymer. This effect has been exploited to enhance the chemical stability of the loaded curcumin. According to the instability of Cur-Nano3 when kept at 4 °C, it was considered that the higher aromatic substitution in Cur-Nano3 led the system to have higher hydrophobicity. At the same time, the storage temperature also showed the influence of this nanoformulation system on the solubility. The decrease in storage temperature caused a decrease in the solubility of the system. These effects therefore influenced the size and caused some precipitation of Cur-Nano3.

## 4. Conclusions

This present study demonstrates that the stabilization effect of curcumin in the polymeric micelles depends on the substituted side chain in the polymer. Hydrolysis of the side chain group caused the nanoformulation to have more hydrophilicity and swelling properties. The aromatic substitution led to an increase in hydrophobicity and the π-π stacking interactions between the aromatic groups of the polymers and curcumin led to higher stability. The Cur-Nano with the highest content of aromatic groups is underlined as a promising nanoformulation with good storage stability for the solubilization and stabilization of curcumin.

## Figures and Tables

**Table 1:** Loading of curcumin in polymeric micelles.

Formulation	Aromatic Content in Polymer (%)	EE (%)	LC (%)
Cur-Nano1	0	61.3 ± 0.5	6.8 ± 0.1
Cur-Nano2	25	61.3 ± 0.1	6.8 ± 0.0
Cur-Nano3	100	113.8 ± 3.4	12.6 ± 0.4

Cur-Nano: curcumin nanoformulation; EE: entrapment efficiency; LC: loading capacity.
